# The Metabolomic Rationale for Treating Perimenopausal Syndrome as Kidney Deficiency

**DOI:** 10.1155/2020/8568353

**Published:** 2020-12-09

**Authors:** Xueqin Chen, Caiming Wu, Wen'na Liang, Jianying Shen, Zewei Zhuo, Liu Hu, Luwei Ruan, Pengheng Zhang, Leqin Xu, Chengfu Li, Shengyuan Lin, Junjie Lan, Haixia Ren, Hongwei Yao, Tongjin Zhao, Bizhen Gao, Tianwei Lin, Huiying Huang, Candong Li

**Affiliations:** ^1^The First Affiliated Hospital of Xiamen University, Xiamen, Fujian 361026, China; ^2^Xiamen Hospital of Traditional Chinese Medicine Affiliated to Fujian University of Traditional Chinese Medicine, Xiamen, China; ^3^State Key Laboratory of Cellular Stress Biology, Innovation Center for Cell Signaling Network, State-province Joint Engineering Laboratory of Targeted Drugs from Natural Products, School of Life Sciences, Xiamen University, Xiamen, Fujian, China; ^4^Cancer Research Center of Xiamen University, Xiamen, Fujian, China; ^5^Fujian University of Traditional Chinese Medicine, Fuzhou, China; ^6^First Hospital Affiliated to Fujian University of Medicine, Fuzhou, China; ^7^School of Chemistry and Chemical Engineering, Xiamen, Fujian, China

## Abstract

**Background:**

Traditional Chinese medicine (TCM) typically attributes the etiopathogenesis of perimenopausal syndrome (PMS) to kidney deficiency in the TCM stratification system for diagnosis. However, the molecular basis of this classical attribution remains to be investigated. *Aim of the Study*. By unraveling the responses to TCM treatment for kidney deficiency, the metabolomic link between PMS and kidney deficiency can be evaluated for in-depth understanding of the mechanism of TCM treatment and development of better treatment protocols.

**Materials and Methods:**

With naturally aged rats as a model for PMS, the metabolomic response to TCM treatment for kidney deficiency was investigated by ^1^H NMR.

**Results:**

^1^H NMR metabolomic evidence of plasma samples demonstrates that treatments with two classical TCM prescriptions for kidney deficiency, decoctions of Yougui and Zuogui, result in modulating the metabolic state of the disease model towards that of rats of younger age.

**Conclusion:**

The data support the notion that kidney deficiency is responsible, in part at least, for PMS, and the relevant prescriptions are helpful in dampening the changes in the body's metabolic states to alleviate symptoms of the disorder.

## 1. Introduction

Perimenopause is a transition state towards women's reproductive senescence. With the declining level of estrogens, the estrogen receptor network is being decoupled from multiple signaling and transcriptional pathways during the process [1–4]. As the body undergoes profound changes, perimenopause is highly symptomatic to women as many experience hot flushes, insomnia, night sweats, and urogenital atrophy, which have been collective called perimenopausal syndrome (PMS). Not only PMS downgrades the quality of life, it could also lead to age-related cardiovascular diseases, osteoporosis, neurological disorder, and other disorders [1, 3, 5–7].

TCM has been practiced for treating PMS for centuries. The practice of TCM follows its symptom-based diagnostic system, the pattern differentiation. Pattern differentiation (also translated as “syndrome differentiation,” “Zheng differentiation,” “pattern diagnosis,” and “pattern classification”), a syndrome stratification according to TCM diagnostic approach, plays a central role in the theory and practice of TCM [8–11]. Typically PMS is diagnosed in the system of pattern differentiation as kidney deficiency [12]. It has long been established that the decoction of Zuogui (Zuogui) and the decoction of Yougui (Yougui) are two effective prescriptions for treating kidney deficiency.

Metabolic profiles are important indicators of physiological and pathological states. As the intermediates and products of metabolism, metabolites could be identified and quantified by metabolomics for chemical fingerprints of cellular processes perturbed by disease and treatment. Metabolite profiles generated by the algorithms of metabolomics are important indicators of metabolic states and provide an avenue to identify biomarkers of treatment efficacy [13]. The comparative profiling of metabolites after treatment with these two decoctions can shed light on the practice of TCM based on pattern differentiation. Herein, naturally aged and perimenopausal rats were used as a PMS model. The metabolite profiles of plasmas after treatments with Yougui and Zuogui were compared based on ^1^H NMR metabolomics. The results showed that both decoctions are applicable for treating PMS by attenuating the metabolic states of the treated. The data also indicate that, while kidney is the root cause, other factors might also play a role in PMS.

## 2. Materials and Methods

### 2.1. Preparing the Extracts of Yougui and Zuogui

The Chinese herbal ingredients for Yougui and Zuogui are listed in Tables 1 and 2. The individual herbs that constituted Yougui and Zuogui were purchased from the Third Peoples Hospital of Fujian University of Traditional Chinese Medicine (FJUTCM; Fuzhou, China) and were identified by the Teaching and Research section of FJUTCM.

The decoctions were prepared by the formula in the Chinese Pharmacopoeia, version 2015. The ingredients were mixed and soaked in water (1 : 8 w/v) for 0.5 hours, which were then boiled for 1.5 hours and filtered. Water (1 : 6 v/w) was added again to the herbal mixes and boiled for 1.5 hours. The filtrates were combined and stored at 4°C. The decoctions were warmed to room temperature for gavage.

### 2.2. Generation of a PMS Model with Naturally Aged Rats

All rats were treated following the Guidelines for the Care and Use of Laboratory Animals from the National Institute of Health. The experiments were approved by the Animal Care and Use Committee of the Fujian University of Traditional Chinese Medicine. All surgical procedures were carried out under sodium pentobarbital anesthesia, and every effort was made to minimize suffering.

SPF grade female Sprague–Dawley rats of 3 months old were acquired from Beijing Vital River Laboratory Animal Technology Co. in Beijing, China. The animals were housed with a controlled humidity of 55% and temperature of 23°C with a 12-hour light/dark cycle. A normal food and water supply was also provided. Swaps from the vagina were taken everyday when the rats reached the age of 11 months old and continued for at least 8 days for microscopic observations of shedding cells. The rats were deemed to be perimenopausal when the menstrual cycle became irregular typically at the ages of about 11–13 months old [14, 15]. The appearance, weight, food intake, and activity were monitored throughout the experiment. Estrogen (E2) concentrations in the plasma were estimated by ELISA.

The decoctions were applied by gavage once daily for 4 weeks with a dose of 1 mL for every 100 g of body weight. After an overnight fasting, blood samples were taken at each period and centrifuged. The plasmas were collected and stored at −80°C.

### 2.3. Sample Collection and NMR Data Acquisition

^1^H NMR spectra were acquired in a Bruker 600 MHz AVANCE II NMR spectrometer (Bruker Biospin, Rheinstetten, Germany) operating at a 1H frequency of 600.13 MHz and a temperature of 298 K. A one-dimensional (1D) Carr–Purcell–Merboom–Gill (CPMG, RD-90-(*τ*cp-180-*τ*cp)-acquisition) with water suppression and a total spin–spin relaxation delay of 320 ms was used to attenuate the broad signals from proteins and lipoproteins due to their short transverse relaxation time. The ^1^H NMR spectrum for each sample consisted of 80 scans with the following parameters: spectral width = 12335.5 Hz, spectral size = 65,536 points, pulse width (PW) = 30° (12.7 *μ*s), and relaxation delay (RD) = 2.0 s. The FIDs were Fourier transformed with LB = 0.3 Hz.

The ^1^H NMR spectra were manually phased, and the baseline was set by using the software Topspin 3.2. Integrations of water resonance (4.70–5.15 ppm) in the spectra of aqueous samples were excluded. The metabolites were normalized to the total integrated spectral area (−0.55–8.55 ppm) for aqueous samples. The dataset was log-transformed and Pareto-scaled (mean-centered and divided by the square root of the standard deviation of each variable) before statistical analysis.

### 2.4. Multivariate Analysis

The multivariate analyses were carried out by the algorithm of PLS-DA implemented in MetaboAnalyst 4.0 [16]. The parameters for model fitness (*R*^2^) and predictive ability (*Q*^2^) were used to judge the quality of the model. A model with a large *R*^2^ (close to 1) and *Q*^2^ (*Q*^2^ ≥ 0.5) is considered acceptable. The validation of the PLS-DA model was also carried out by the permutation test in which the class membership was randomly shuffled by 100 times for calculating the response values. The new *R*^2^ and *Q*^2^ values were lower than the original ones indicating no overfitting [17].

Important metabolites were identified by a variable influence on the projection (VIP) score in the PLS-DA analysis. A *t*-test was used to evaluate the significant differences in the selected signals of the main metabolites that were responsible for class discrimination using GraphPad Prism 5. The data are presented as *p* < 0.05(*∗*), *p* < 0.01(*∗∗*), and *p* < 0.001(*∗∗∗*).

## 3. Results and Discussion

### 3.1. Generating the Rat Models

**Figure 1:**
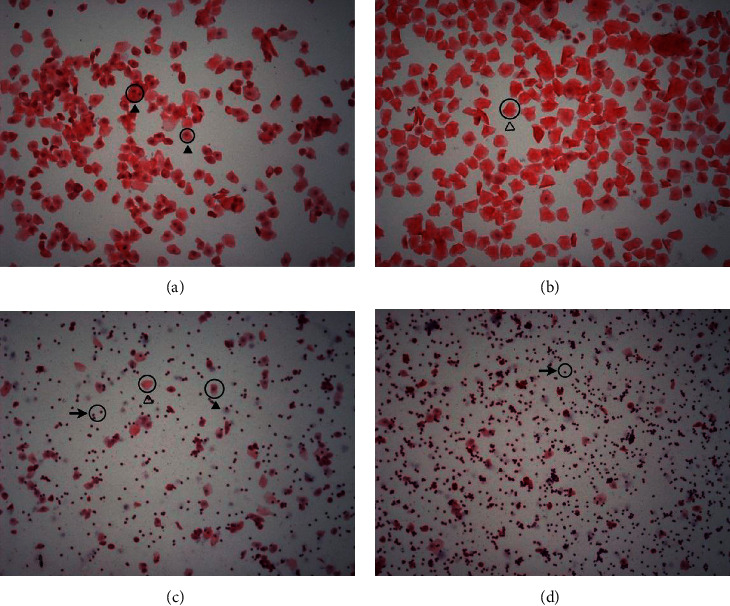
Establish a model for perimenopausal syndrome. (a) Swaps from the vagina of rats in the proestrus stage were mainly with epithelial cells with few keratinocytes. (b) There were mainly keratinocytes in the swaps from the vagina of rats in the estrus stage. (c) Roughly equal amount of epithelial cells, keratinocytes, and leucocytes were visible for rats in the metestrus stage. (d) The rats in the diestrus stage were mainly with leucocytes and with few epithelial cells and keratinocytes in the swaps from the vagina.

SPF grade Sprague–Dawley rats of 3 month old were acquired, raised, and checked for signs of perimenopause with the microscopic examination of the cells shedding in vagina (Figure 1). Rats in the proestrus stage were mainly with epithelial cells and few keratinocytes in the swaps from the vagina (Figure 1(a)). Rats in the estrus stage were mainly with keratinocytes (Figure 1(b)). Roughly equal amount of epithelial cells, keratinocytes, and leucocytes were in the vagina swaps for rats in the metestrus stage (Figure 1(c)). The rats in the diestrus stage were mainly with leucocytes and with few epithelial cells and keratinocytes (Figure 1(d)). Rats without the estrum in five consecutive days were considered perimenopausal. The averaged estrogen (E2) in the plasma was lower at 4.9 ± 0.6 pg/mL for the perimenopausal rats, while the plasma E2 concentration for the rats of 6 months old was 6.9 ± 1.1 pg/mL (Table 3). There was no significant weight change after the rats entered the phase of perimenopause (Table 4).

### 3.2. NMR Data Acquisition and Analysis

The plasma samples from the rat model were obtained after an overnight fasting and analyzed by ^1^H NMR (Figure S1). The characteristic spectra for each metabolite in the plasma were interpreted based on the Human Metabolome Database [18]. Over 30 metabolites were identified and quantified for analysis with the algorithm of PLS-DA implemented in MetaboAnalyst 4.0 [16].

### 3.3. Perimenopause Concomitant with Altered Metabolic Profiles

**Figure 2:**
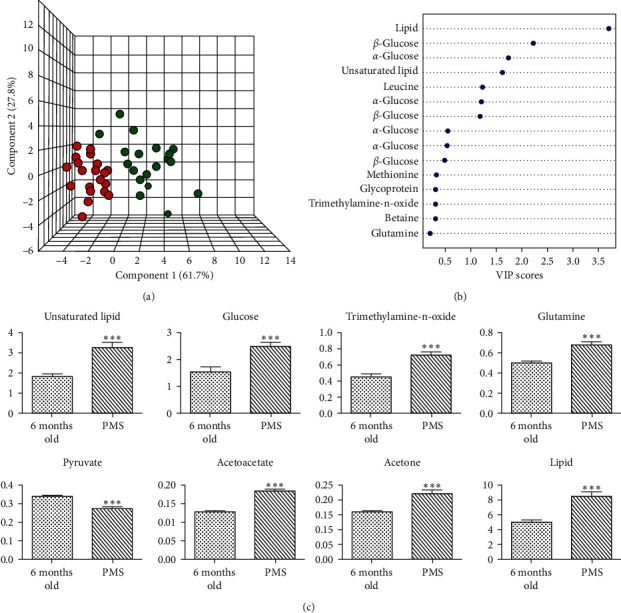
The metabolic profile of perimenopausal rats. (a) 3D representation of PLS-DA analysis of those just entering the perimenstrual period (green) and those that have not entered the perimenopausal period with younger age (red). The rats entering the perimenopausal period were with distinctly different metabolic profiles over those that were not. (b) Lipids, glucose, and leucine are the metabolites with VIP scores larger than 1, which are all involved in the energy metabolism. (c) The dominant metabolites in the metabolic profile for rats entering the perimenopausal period are those for the energy metabolism and amino acids. ^*∗∗∗*^*p* < 0.001.

**Figure 3:**
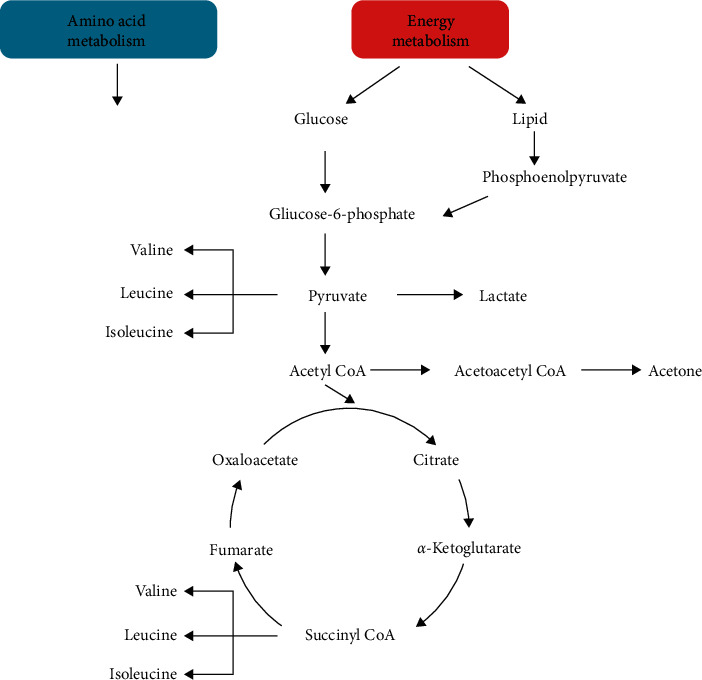
Major metabolites in the energy metabolism. Estrogen receptor system regulates the energy metabolism as a part of its regulation for reproduction. With the declining levels of estrogen during perimenopause, the body goes through a drastic change by decoupling the estrogen receptor system from the energy metabolism.

The NMR spectra from the plasmas of rats that just entered the perimenopause at 11–13 months old and those at 6 months old were analyzed. The metabolic profiles from those entering the perimenopausal state and those at younger age were clearly different (Figure 2(a)), indicating that they were of different metabolic states. VIP scores showed that the dominant metabolites in the metabolomes were lipids, glucoses, and leucine (Figure 2(b)). Other important metabolites include trimethylamine-n-oxide, betaine, citrate, glutamine, pyruvate, acetoacetate, acetone, acetate, and valine (Figure 2(c), Figure S2, and Table S1). Among these metabolites, the majority is for energy metabolism, such as lipid, glucoses, citrate, glutamine, pyruvate, valine, leucine, acetoacetate, acetone, acetate, and betaine [19] (Figure 3). There are also indications of metabolism of amino acids, such as methionine and glutamine. Glutamine is also an energy source, next to glucose [20]. The increased amount of trimethylamine-n-oxide in the plasma of perimenopausal rats indicates an increased risk of atherosclerosis [21].

### 3.4. The Metabolic Profile of Perimenopausal Rats after Saline Treatment

**Figure 4:**
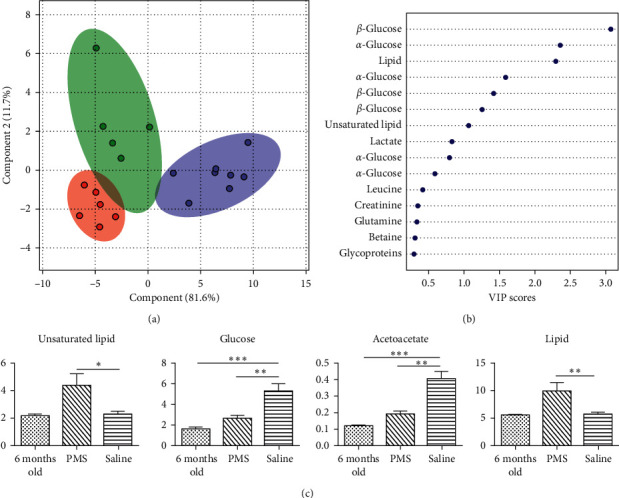
Perimenopause accelerated the metabolic change. (a) PLS-DA analysis of metabolites in the plasma of rats that had not entered the perimenopausal period (red), rats that just entered the perimenopausal period (green), and rats that were treated with saline for 4 weeks (purple). As age progressed, the metabolic patterns drifted further away from that before entering the perimenopause. The metabolomic separation between the green state (perimenopause) and purple state (4 weeks after entering the perimenopause) is greater than the metabolomic separation between the red state (5–7 months before entering the perimenopause) and green state and is an indication that the metabolic change was accelerated after the rats entered the perimenopause phase. (b) As the aging continued, the major metabolites in the metabolic profile were predominantly energy metabolism-related, and at the top of the VIP list were glucose, lipid, and lactate. (c) Four weeks after entering perimenopause and treatment with saline, there were important changes in metabolite quantities, which included lipids, glucose, acetoacetate, and others. An important feature is that the metabolism of lipid was down, while that of glucose was up. ^*∗*^*p* < 0.05; ^*∗∗*^*p* < 0.01; ^*∗∗∗*^*p* < 0.001.

A group of perimenopausal rats were treated with normal saline as the treatment control for 28 days. The multivariate statistical analysis showed that the metabolomes of the saline-treated perimenopausal rats drifted further away from that in the initial phase of the perimenopause (Figure 4(a) and Figure S3). The metabolomic separation of rats at 6 months old and rats that just became perimenopausal at 11–13 months old was smaller than the metabolomic separation of rats that just entered the phase of perimenopause and rats treated with saline for 4 weeks (Figure 4(a)). It is an indication that the change of the metabolic state was accelerated after the rats entered the phase of perimenopause.

Four weeks into the perimenopause, the major metabolites in the metabolic profile are predominantly for energy metabolism, such as glucose and lipid (Figure 4(b)). The other important metabolites are lactate, creatine, betaine, creatinine, citrate, glutamine, succinate, and acetoacetate. An important feature in this change is that the lipid metabolism was downregulated and the glucose-related metabolism was upregulated (Figure 4(c), Figure S4, and Table S2), but there was little change in the bodyweight (Table 4). The elevated levels of metabolites such as acetoacetate indicate that the system is responding to stress.

### 3.5. Metabolomic Improvements by Treating Perimenopausal Rats with Yougui

PMS are normally associated with the symptoms of kidney deficiency by TCM, and centuries of practice show that prescribing Chinese herb formulas for treating kidney deficiency can alleviate the symptoms of PMS. Kidney deficiency could be further classified into yin and yang types in the strata of pattern differentiation. The two subtypes of kidney deficiency can also be concurrent as the kidney yin/yang deficiency [22–24]. Kidney yin deficiency increasingly occurs in middle-aged women from premenopausal to perimenopausal years and is common in women with menopausal hot flushes [25]. Kidney yang deficiency generally occurs later in the process of reproductive senescence and frequently in the postmenopausal years. Kidney yin/yang deficiency is common in menopausal women [26, 27].

**Figure 5:**
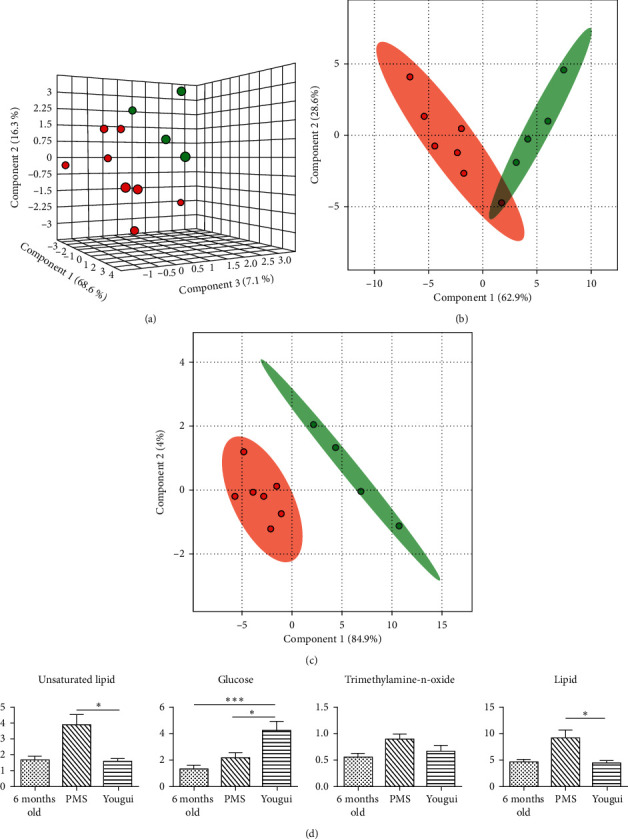
Treatment with Yougui. (a) Yougui altered the metabolic profile of perimenopausal rats. The red dots were from rats treated with saline, while green ones were from rats treated with Yougui. The two groups of rats were of the same age, and the difference in PLS-DA analysis indicated that Yougui perturbed the metabolism of rats. (b) PLS-DA analysis of metabolites from the plasma of Yougui-treated rats (green) in reference to that of rats in the early phase of perimenopause (red) indicated that Yougui modulated the metabolic profile of rats towards that of rats at younger age. (c) The metabolome of the Yougui-treated rats (green) was still distinct from that of rats that had not entered the perimenopausal period (red). (d) The level of lipid was significantly downregulated by Yougui to the levels of rats at 6 months old. The level of glucose and other metabolites in the TCA cycle was significantly upregulated. Importantly, the plasma level of trimethylamine-n-oxide was lowered to that of rats of much younger age. ^*∗*^*p* < 0.05; ^*∗∗∗*^*p* < 0.001.

The classical prescription for kidney yang deficiency is Yougui. Yougui has been proven effective in treating kidney yang deficiency through centuries of TCM practice. Perimenopausal rats were fed with Yougui once daily for 28 days (Figure 5). It was shown that Yougui-treated rats were with different metabolic profiles from saline-treated rats (Figure 5(a)). Yet unlike the saline treatment, the metabolic profile of Yougui-treated rats, although did not overlap with, was closer to that of rats of younger age at the initial phase of perimenopause (Figure 5(b)). There was no indication though that Yougui treatment would result in bringing the metabolic profile of perimenopausal rats to that of rats before entering the perimenopausal period at the age of 6 months old (Figure 5(c)). Yougui modulated those same metabolites that changed during saline treatment (Figure 5(a)), but obviously Yougui tuned the metabolomic state in a more desirable direction (Figure 5(b)) away from the saline-associated metabolic state (Figures 4(a) and S3). Quantitative analysis showed that the level of plasma lipids was also down, similar to that in the saline-treated group (Figures 5(c) and 5(d) and Table S3). The levels of glucose, citrate, succinate, amino acids, and others were higher in the plasma, but the rises were not as drastic as that in the saline-treated group, implicating that Yougui could moderate changes in the body in response to the decline of estrogens. A distinctive feature in the Yougui treatment is that the level of trimethylamine-n-oxide was downregulated to the level of rats of 6 months old, implicating a reduced risk of cardiovascular diseases [21] (Figures 5(c) and 5(d) and Table S3). Yougui treatment also led to the reduction of weight (Table 4). It can also be shown that Yougui treatment did not result in higher levels of estrogen (Table 3), indicating Yougui could compensate for the decoupling of the estrogen receptor system from the energy metabolism and slow down the changes in the body for the relief of PMS symptoms.

### 3.6. Metabolomic Evidence for Treating PMS as Kidney Yin Deficiency

**Figure 6:**
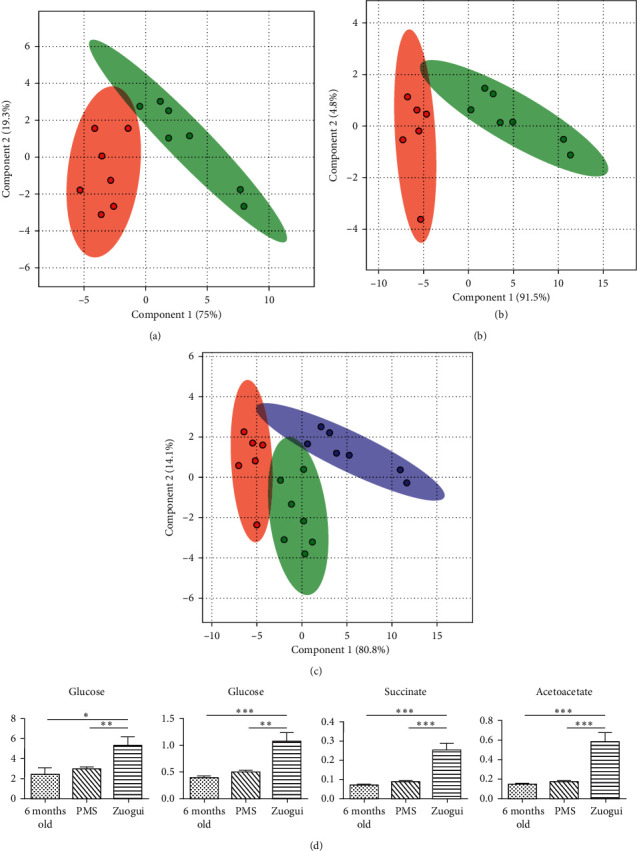
Treatment with Zuogui. (a) Treatment with Zuogui brought the metabolomic pattern of the plasma of perimenopausal rats towards that of rats at the initial phase of perimenopause before treatment. (b) Zuogui even brought the metabolomic profile of the plasma of perimenopausal rats (green) towards that of rats that had not entered the perimenopause at the age of 6 months old (red). (c) Zuogui brought the metabolic state of perimenopausal rats towards that at younger ages. Red for rats that had not entered the perimenopausal period, green for perimenopausal rats, and purple for Zuogui-treated rats. (d) Zuogui modulates many metabolites in the energy metabolism at moderately higher levels. The exceptions were succinate and acetoacetate, which are elevated to significantly higher levels in the plasma. ^*∗*^*p* < 0.05; ^*∗∗*^*p* < 0.01; ^*∗∗∗*^*p* < 0.001.

Paralleling to Yougui, TCM prescription for kidney yin deficiency is Zuogui. For the perimenopausal rats, Zuogui modulated the metabolic profile not only towards that in the initial phase of the perimenopause but also brought the metabolic state close to that before entering the perimenopause (Figures 6(a)–6(c)), without elevating the plasma estrogen level (Table 3). Zuogui downregulated the levels of lipid and upregulated the level of glucose, but more moderately, as compared to the Yougui-associated regulation (Figures 6(d) and S6 and Table S4). The body weight also seemed to be reduced (Table 4). Many metabolites in the energy metabolism and amino acids are also of moderately higher levels. The exceptions were elevated concentrations of succinate and acetoacetate over other metabolites in the plasma, indicating active TCA cycle. Higher level of activity in the energy metabolism is an indication of overcoming kidney deficiency (Figures 6(d) and S6 and Table S4).

## 4. Discussion

Perimenopause is a transition state in the midlife of women towards reproductive senescence. As multiple levels of the cellular process are undergoing adjustment with the declining level of estrogen, fundamental changes in the body could lead to PMS. TCM offers an avenue to smooth the process, and centuries of practice attest its effectiveness in treating PMS.

In the strata of pattern differentiation of the TCM diagnostic system, PMS is frequently attributed to kidney deficiency and treated with prescriptions such as Yougui and Zuogui. Our data suggest that both Yougui and Zuogui were of benefits for treating PMS in the metabolic level. In particular, Zuogui seems to be a more balanced treatment and could blunt the drastic changes in the metabolic state of perimenopausal rats during the transition to reproductive senescence, while Yougui treatment would have the benefit of downregulating the level of trimethylamine-n-oxide in plasma with the prospect of reducing the risk of cardiovascular diseases. Zuogui's tuning of the metabolic profile seems to be more effective than Yougui's in perimenopausal rats, which might reflect the fact that the animal model was rats at their early stage of perimenopause when kidney yin deficiency would be more prevalent. However, that Yougui also perturbed the metabolic state of the rat model towards the more desirable direction, albeit at a lesser degree as compared to Zuogui, is an indication that the naturally perimenopausal rats generated with our protocol might actually model the kidney yin/yang deficiency with a predominant yin.

Despite the fact that both Yougui and Zuogui modulate metabolic profiles of perimenopausal rats towards that of rats at younger age, the overall effect is modest. There are three possible causes for this phenomenon. First, the TCM treatment tends to be mild and generally fine-tunes body functions to heal. Second, PMS might not be adequately modeled with naturally aged rats. Rats are of different pathophysiological characteristics to human and can only partially model the human diseases. The humanly yin/yang system might not be properly modeled in rats with a distinct energetic level. The emotional aspect of PMS could not be modeled in rats either. Third, the etiopathogenesis of PMS might be more than just kidney deficiency. PMS is age-related, which argues for aging-associated kidney deficiency that results in PMS. However, PMS would mostly diminish naturally with the pass of time while the patients are not getting younger. Clearly, kidney deficiency is the precondition for PMS but cannot be fully accounted for PMS in many cases. In the modern practice of TCM, some PMS cases have been attributed to liver qi stagnation in concurrence with kidney deficiency in pattern differentiation [12, 28]. It is of great importance to reveal biomarkers to better stratify PMS cases as either kidney deficiency, liver qi stagnation, or a combination of both so that more effective treatments could be prescribed in the future studies with suitable animal models.

## 5. Conclusions

We show here that perimenopause is coupled with metabolomic shifts predominantly resulting from changes in the energy metabolism. Metabolomics provide direct functional readouts of the physiological states. The fact that Zuogui and Yougui, TCM prescriptions for treating kidney deficiency, could modulate the metabolism of rat models towards more favorable states in this metabolomic investigation provides solid evidence to support treating perimenopause as kidney deficiency.

## Figures and Tables

**Table 1:** Herbal composition of Yougui.

Chinese name	Botanical name	Common name	Ratio

Shu Di Huang	*Rehmannia glutinosa* (Gaertn.) DC.	Rehmanniae Radix Praeparata	3
Shan Yao	*Dioscorea oppositifolia* L.	Dioscorea rhizome	3
Shan Zhu Yu	*Cornus officinalis* Siebold & Zucc.	Corni Fructus	2
Gou Qi Zi	*Lycium barbarum* L.	Lycii Fructus	3
Gan Cao	*Glycyrrhiza uralensis* Fisch	Glycyrrhizae Radix et Rhizome	1
Du Zhong	*Eucommia ulmoides* Oliv.	Eucommiae Cortex	3
Rou Gui	*Cinnamomum cassia* (L.) J.Presl	Cinnamomi Cortex	1
Fu Zi	*Aconitum carmichaelii* Debeaux	Aconiti Lateralis Radix Praeparata	2

**Table 2:** Herbal composition of Zuogui.

Chinese name	Botanical name	Common name	Ratio

Shu Di Huang	*Rehmannia glutinosa* (Gaertn.) DC.	Rehmanniae Radix Praeparata	6
Shan Yao	*Dioscorea oppositifolia* L.	Dioscorea rhizome	4
Shan Zhu Yu	*Cornus officinalis* Siebold & Zucc.	Corni Fructus	4
Gou Qi Zi	*Lycium barbarum* L.	Lycii Fructus	4
Zhi Gan Cao	*Glycyrrhiza uralensis* Fisch	Glycyrrhizae Radix et Rhizome Praeparata cum Melle	2
Fu Ling	*Poria cocos* (Schw.) Wolf	Poria	3

**Table 3:** Estrogen (E2) concentrations.

Rats	E2 (pg/mL)
6 months old	6.9 ± 1.1
Perimenopausal	4.9 ± 0.6
Yougui-treated	3.8 ± 0.5
Zuogui-treated	4.3 ± 0.6

**Table 4:** Weight changes (g).

Group	7 months old	Perimenopausal^+^	After treatment^+^

No treatment	330.67 ± 18.61	344.56 ± 19.08	347.67 ± 20.19
Saline-treated	347.13 ± 30.32	343.75 ± 36.46	343.75 ± 27.78
Yougui-treated	330.20 ± 21.05	340.60 ± 21.31	320.80 ± 27.99
Zuogui-treated	339.83 ± 34.87	334.33 ± 27.13	327.83 ± 23.95

^+^4 weeks after the blood samples were taken.

## Data Availability

The data used to support the findings of this study are available from the corresponding author upon request.

## Supplementary Materials

Supplementary Materials Figure S1: identifying metabolites by ^1^H NMR. Figure S2: the dominant metabolites in the metabolic profile for rats entering the perimenopausal period are those for energy metabolism and amino acids. Figure S3: as the aging process continued, the metabolic pattern drifted further away from that of younger rats. Figure S4: metabolites modulated by saline. Figure S5: treatment with Yougui for kidney yang deficiency. Figure S6: metabolites modulated by Zuogui. Table S1: changing metabolites in perimenopause. Table S2: changing levels of metabolites after saline treatment. Table S3: metabolites with changing concentration after Yougui treatment. Table S4: concentration variations after Zuogui treatment.
