# Supplementary material for: The Metabolomic Rationale for Treating Perimenopausal Syndrome as Kidney Deficiency
Source: Evid Based Complement Alternat Med. 2020 Dec 9;2020:8568353. doi: 10.1155/2020/8568353 (PMC7746443; doi:10.1155/2020/8568353)
Supplement: Supplementary Materials — Figure S1: identifying metabolites by 1H NMR. Figure S2: the dominant metabolites in the metabolic profile for rats entering the perimenopausal period are those for energy metabolism and amino acids. Figure S3: as the aging process continued, the metabolic pattern drifted further away from that of younger rats. Figure S4: metabolites modulated by saline. Figure S5: treatment with Yougui for kidney yang deficiency. Figure S6: metabolites modulated by Zuogui. Table S1: changing metabolites in perimenopause. Table S2: changing levels of metabolites after saline treatment. Table S3: metabolites with changing concentration after Yougui treatment. Table S4: concentration variations after Zuogui treatment. [file 8568353.f1.docx]

## Supplementary Materials


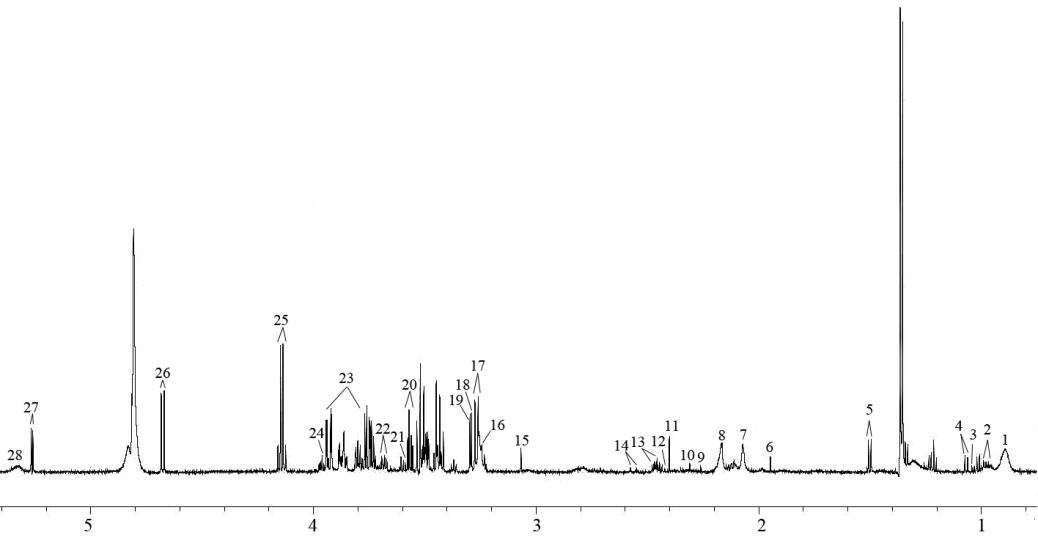


**Fig. S1. Identifying metabolites by ^1^H NMR.** A typical 1H NMR spectrum of rat plasma. Over 30 metabolites were identified based on the Human Metabolome Database, which include (1) lipid; (2) leucine; (3) isoleucine; (4) valine; (5) alanine; (6) acetate; (7) glycoproteins; (8) methionine; (9) acetone; (10) acetoacetate; (11) pyruvate; (12) succinate; (13) glutamine; (14) citrate; (15) creatinine; (16) choline; (17) glucose; (18) betaine; (19) trimethylamine-n-oxide ; (20) glucose; (21) glycine; (22) glycerol; (23) glucose; (24) creatine; (25) lactate; (26) β-glucose; (27) α-glucose; (28) unsaturated lipid.

**
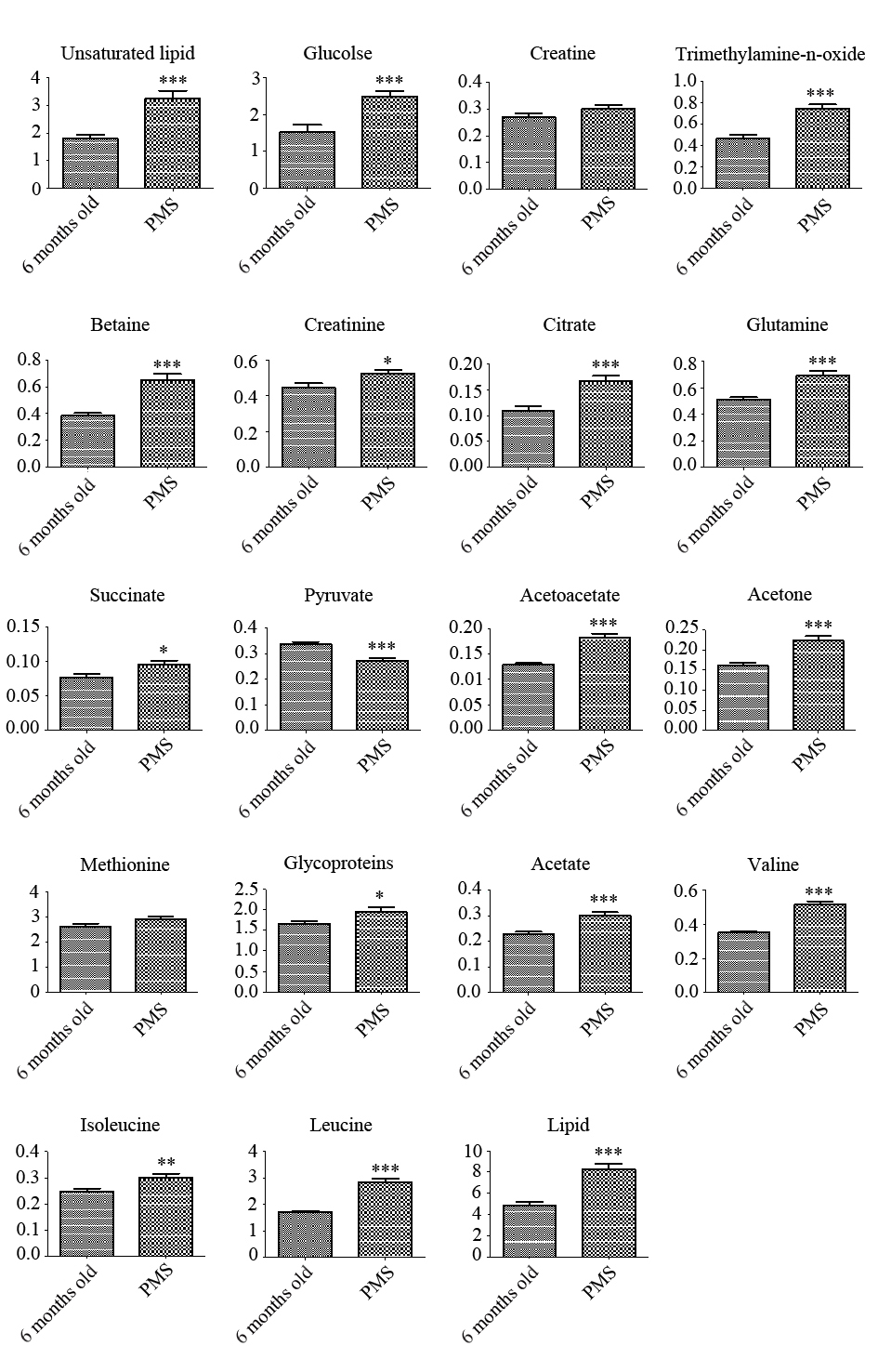
**

**Fig. S2. The dominant metabolites in the metabolic profile for rats entering the perimenopausal period are those for energy metabolism and amino acids.** While the concentrations for unsaturated lipid, glucose, trimethylamine-n-oxide, betaine, creatinine, citrate, glutamine, succinate, acetoacetate, acetone, glyprotein, acetate, valine, isoleucine, leucine, and lipid increase, the concentration for pyruvate was significantly lowered. * p ＜0.05; ** p ＜ 0.01; *** p ＜ 0.001.

**
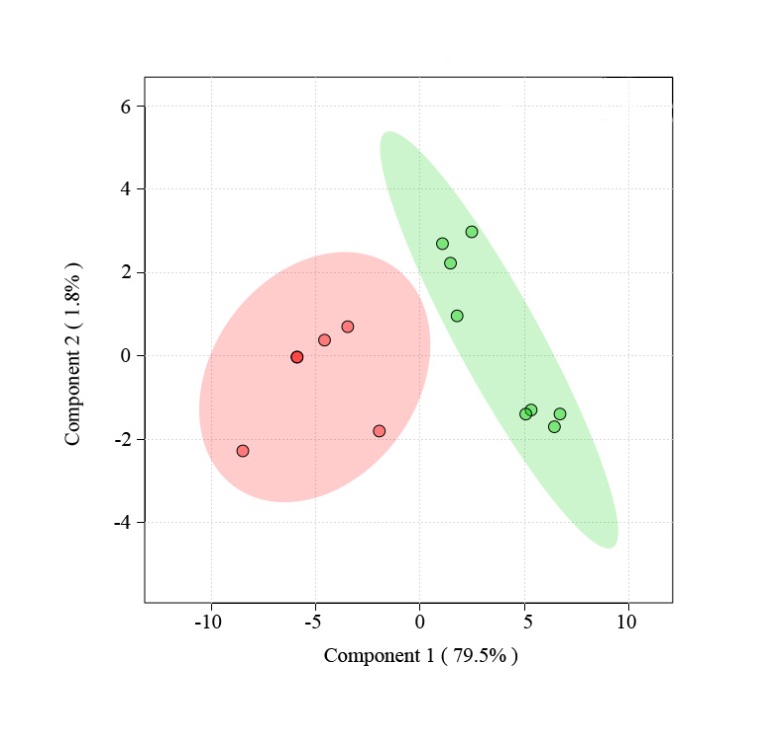
**

**Fig. S3. As the aging process continued, the metabolic pattern drifted further away from that of younger rats.** PLS-DA analysis of saline-treated rats and those just entering the perimenopausal period. The saline-treated rats were 4 weeks older and showed different metabolic profile to those that just entering the perimenopause. Each red dot was from one rat just entering the perimenopausal period. The green dots were from rats 4 weeks after entering perimenopause.


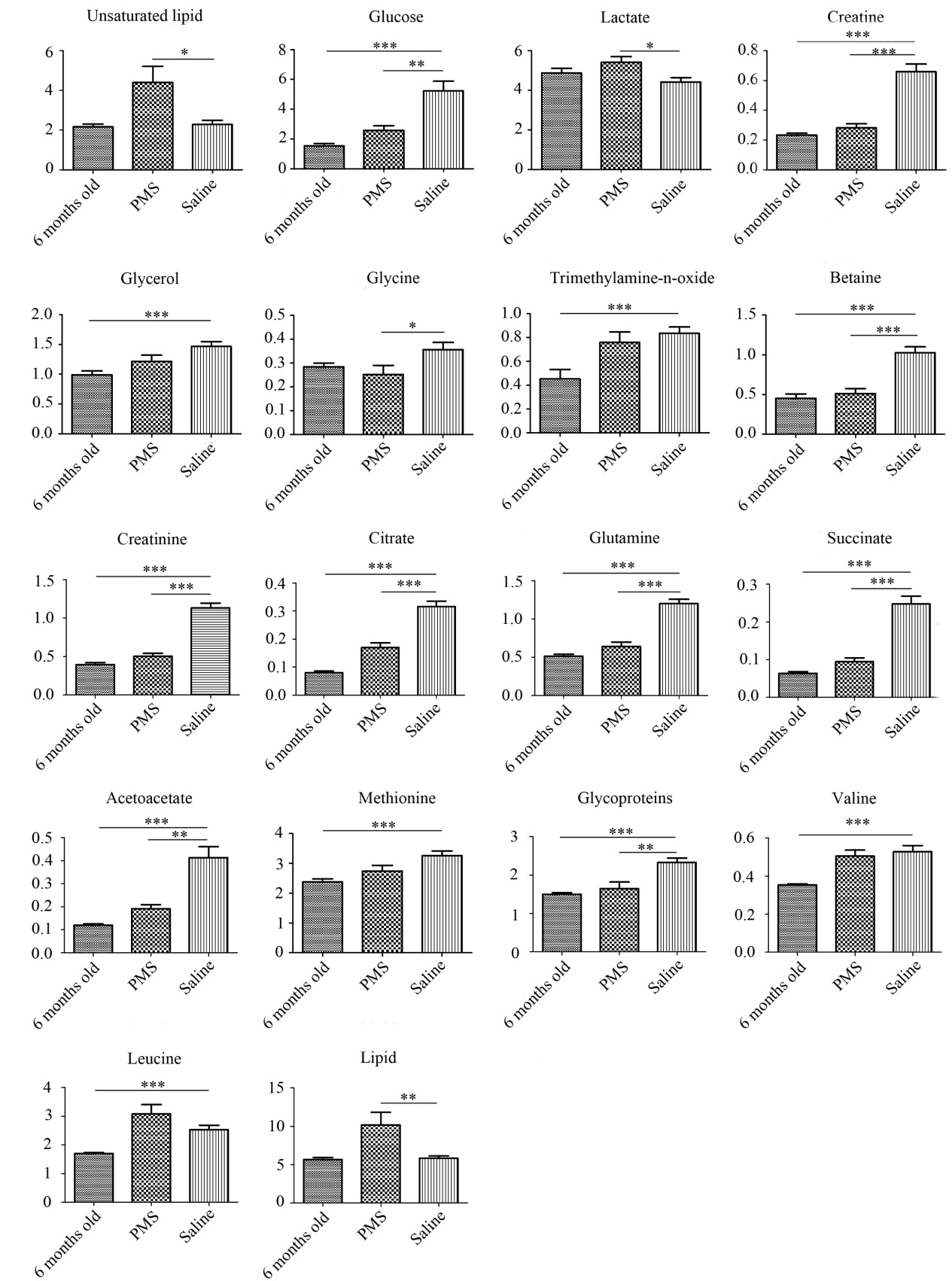


**Fig. S4. Metabolites modulated by Saline.** Four weeks after entering the perimenopause and treatment with saline, there were important changes in the metabolite quantities, which included lipids, glucose, lactate, creatine, citrate, glutamine, succinate, and acetoacetate. An important feature is that the metabolism of lipid was down, while that of glucose was up. * p ＜ 0.05; ** p＜ 0.01; *** p ＜ 0.001.


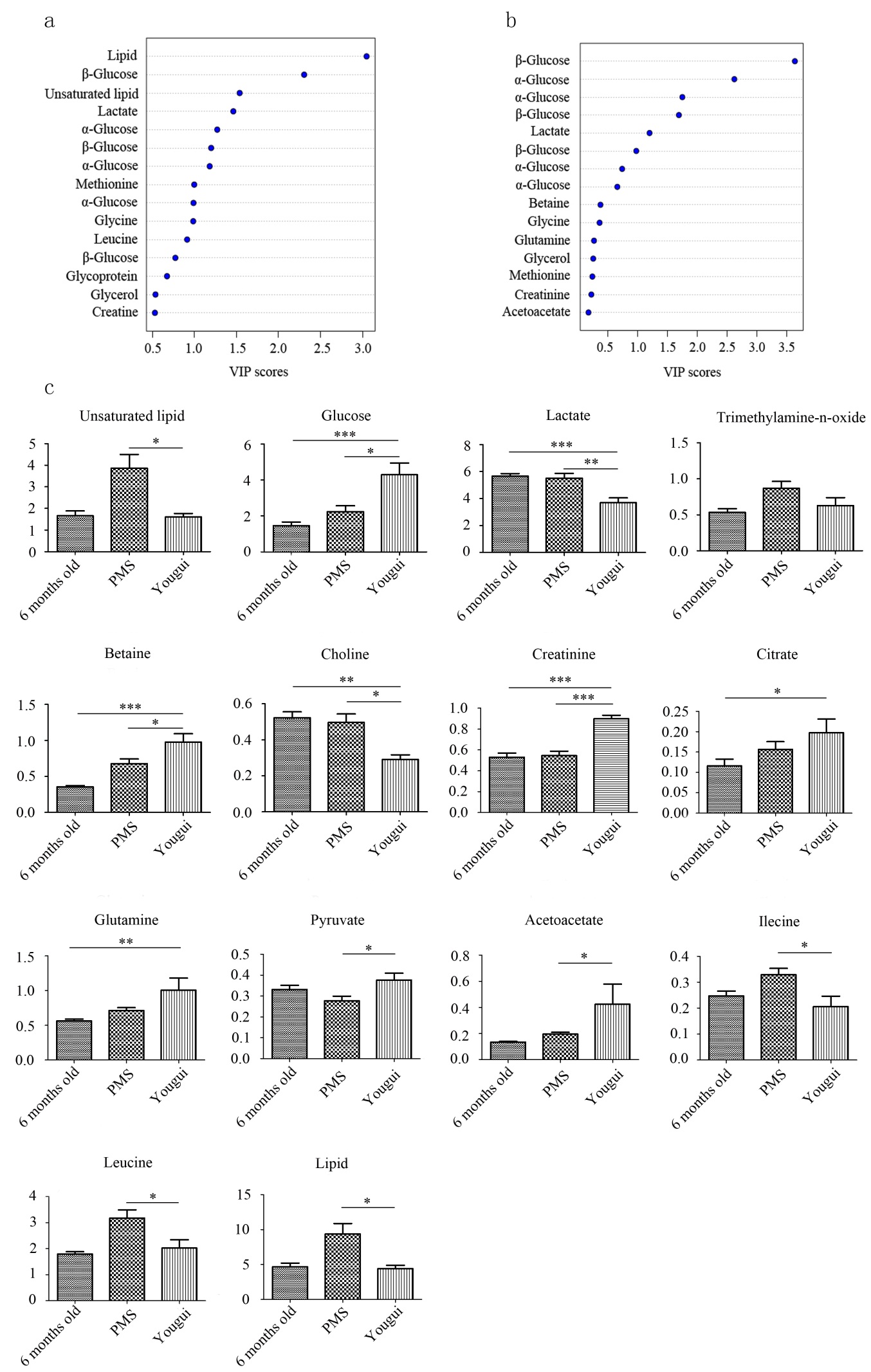


**Fig. S5. Treatment with Yougui for kidney yang deficiency.** (a) In the plasma of Yougui-treated rats, lipids, glucoses, lactate, and amino acids were significantly modulated by the decoction based on VIP scores in comparison with that of saline treated rats. (b) Comparing the metabolites from the plasma of Yougui-treated rats with that of rats that just entered the perimenopause, glucose dominates the metabolome. (c) The level of lipid, as well as acetone and choline, was significantly downregulated by Yougui to the levels of rats at 6 months old. The level of glucose, and other metabolites in the TCA cycle, was significantly upregulated. Importantly, the plasma level of trimethylamine-n-oxide was lowered to that of rats of much younger age. * p ＜ 0.05; ** p ＜ 0.01; *** p ＜ 0.001.

**
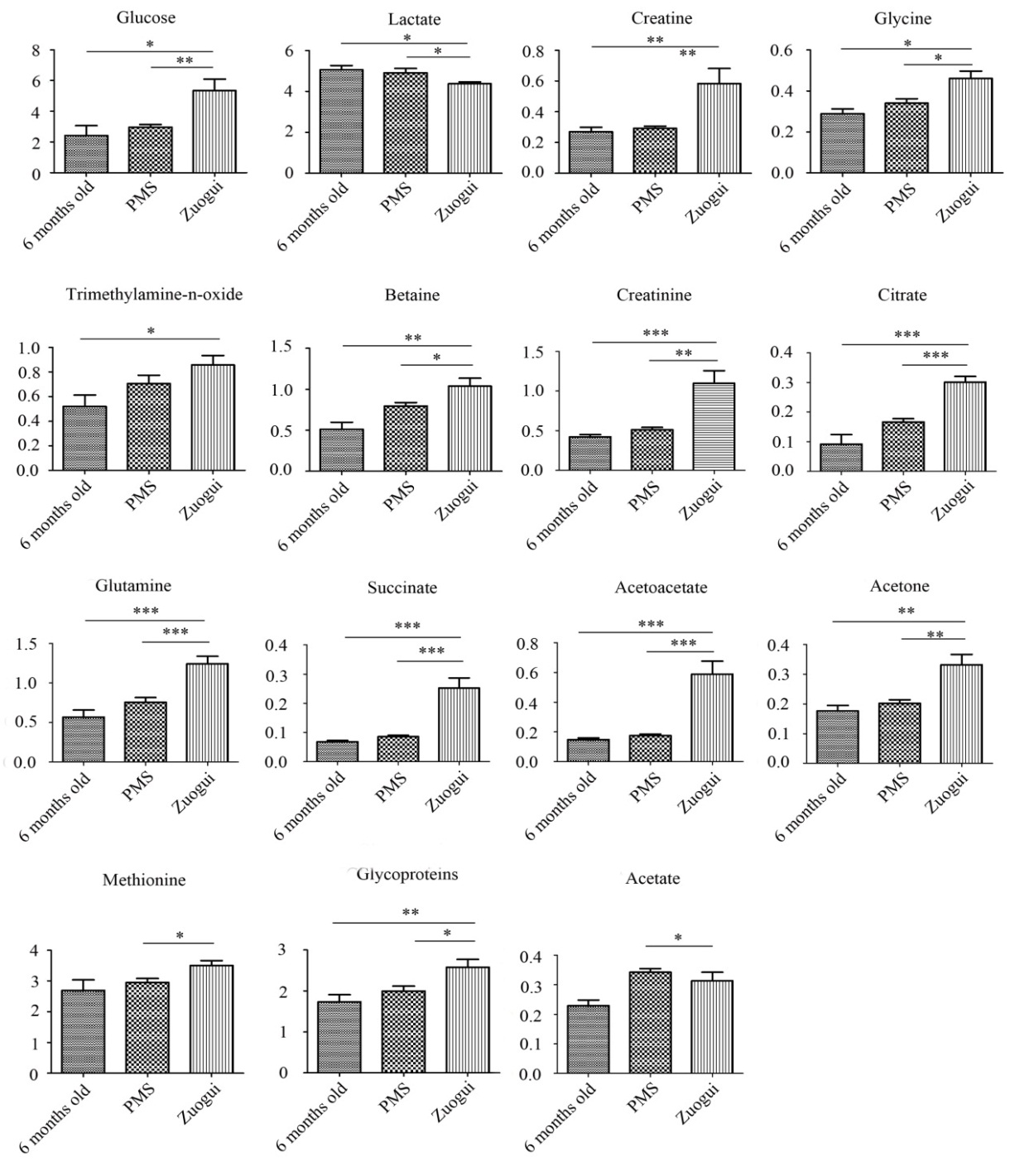
**

**Fig. S6.** **Metabolites modulated by Zuogui.** Zuogui downregulated the levels of lipid and upregulated the level of glucose moderately, as compared to Yougui. Many metabolites in the energy metabolism and amino acids are also at moderately higher levels. The exceptions were succinate and acetoacetate, which are elevated to significantly higher levels in the plasma. * p ＜ 0.05; ** p ＜ 0.01; *** p ＜ 0.001.

Supplementary Tables

Table S1. Changing metabolites in perimenopause

| **Metabolites** | **Perimenopausal *vs* 6 months old rats** |
| --- | --- |
| Unsaturated lipid | ↑*** |
| Glucose | ↑*** |
| Trimethylamine-n-oxide | ↑*** |
| Betaine | ↑*** |
| Creatinine | ↑* |
| Citrate | ↑*** |
| Glutamine | ↑*** |
| Succinate | ↑* |
| Pyruvate | ↓*** |
| Acetoacetate | ↑*** |
| Acetone | ↑*** |
| Glyprotein | ↑* |
| Acetate | ↑*** |
| Valine | ↑*** |
| Isoleucine | ↑** |
| Leucine | ↑*** |
| Lipid | ↑*** |

↑ and ↓ separately represent the increased or decreased metabolite concentrations in the plasma. * p ＜ 0.05; ** p ＜ 0.01; *** p ＜ 0.001.

Table S2. Changing levels of metabolites after saline treatment

| **metabolites** | **mock *vs* 6months old rats** | **mock *vs* perimenopausal rats** |
| --- | --- | --- |
| Unsaturated lipid |  | ↓* |
| Glucose | ↑*** | ↑** |
| Lactate |  | ↓* |
| Creatine | ↑*** | ↑*** |
| Glycerol | ↑*** |  |
| Glycine |  | ↑* |
| Trimethylamine-n-oxide | ↑*** |  |
| Betaine | ↑*** | ↑*** |
| Creatinine | ↑*** | ↑*** |
| Citrate | ↑*** | ↑*** |
| Glutamine | ↑*** | ↑*** |
| Succinate | ↑*** | ↑*** |
| Acetoacetate | ↑*** | ↑** |
| Methionine | ↑*** |  |
| Glycoproteins | ↑*** | ↑** |
| Valine | ↑*** |  |
| Leucine | ↑*** |  |
| Lipid |  | ↓** |

↑ and ↓ separately represent the increased or decreased metabolite concentrations in the plasma. * p ＜ 0.05; ** p ＜ 0.01; *** p ＜ 0.001.

Table S3. Metabolites with changing concentration after Yougui treatment

| **metabolites** | **Yougui-treated *vs* 6months old rats** | **Yougui-treated *vs* perimenopausal rats** |
| --- | --- | --- |
| Unsaturated lipid |  | ↓* |
| Glucose | ↑*** | ↑* |
| Lactate | ↓*** | ↓** |
| Betaine | ↑*** | ↑* |
| Choline | ↓** | ↓* |
| Creatinine | ↑*** | ↑*** |
| Citrate | ↑* |  |
| Glutamine | ↑** |  |
| Acetoacetate | ↑* |  |
| Pyruvate |  | ↑* |
| Isoleucine |  | ↓* |
| Leucine |  | ↓* |
| Lipid |  | ↓* |

↑ and ↓ separately represent the increased or decreased metabolite concentrations in the plasma. * p ＜ 0.05; ** p ＜ 0.01; *** p ＜ 0.001.

Table S4. Concentration variations after Zuogui treatment

| **metabolites** | **Zuogui-treated *vs* 6 months old rats** | **Zuogui-treated *vs* perimenopausal rats** |
| --- | --- | --- |
| Glucose | ↑* | ↑** |
| Lactate | ↓* | ↓* |
| Creatine | ↑** | ↑** |
| Glycin | ↑** | ↑** |
| Trimethylamine-n-oxide | ↑* |  |
| Betaine | ↑** | ↑* |
| Creatinine | ↑*** | ↑** |
| Citrate | ↑*** | ↑*** |
| Glutamine | ↑*** | ↑*** |
| Succinate | ↑*** | ↑*** |
| Acetoacetate | ↑*** | ↑*** |
| Acetone | ↑** | ↑** |
| Methionine |  | ↑* |
| Glycoproteins | ↑** | ↑* |
| Acetate | ↑* |  |

↑ and ↓ separately represent the increased or decreased metabolite concentrations in the plasma. * p ＜ 0.05; ** p ＜ 0.01; *** p ＜ 0.001.
